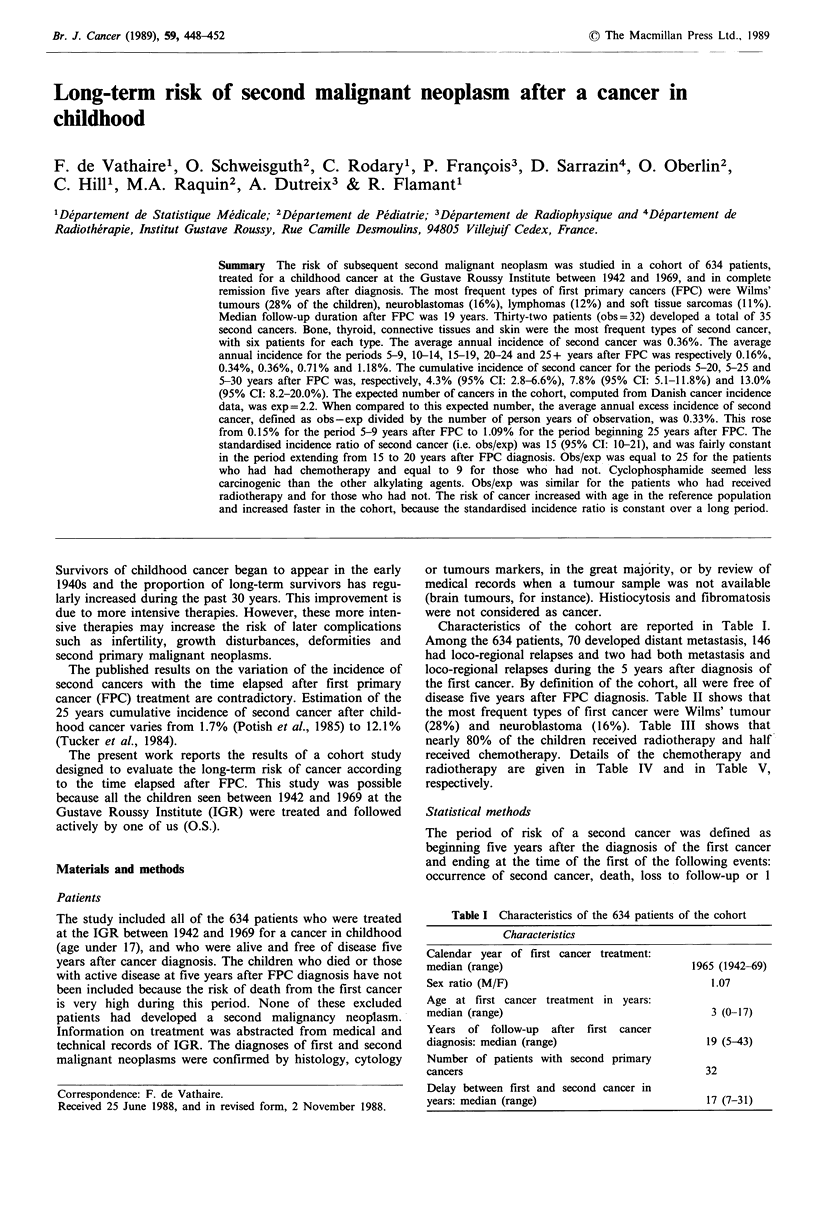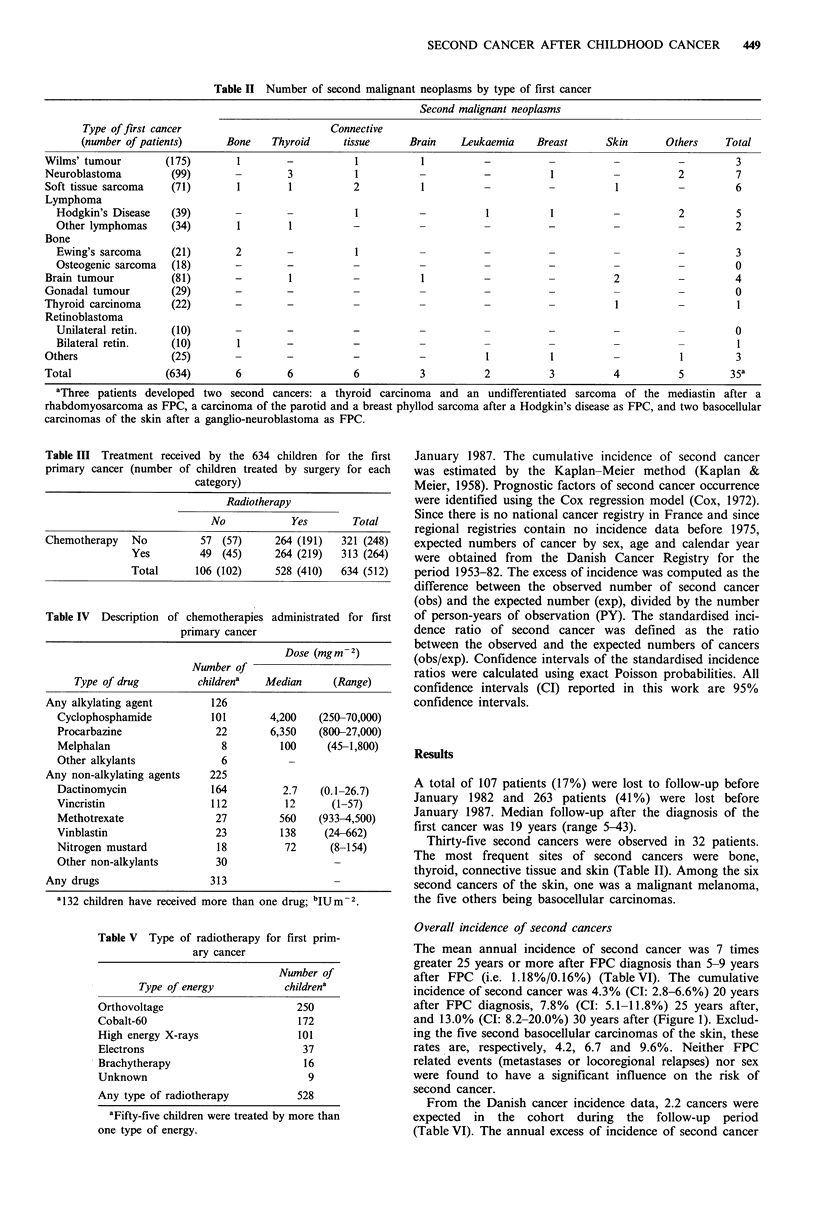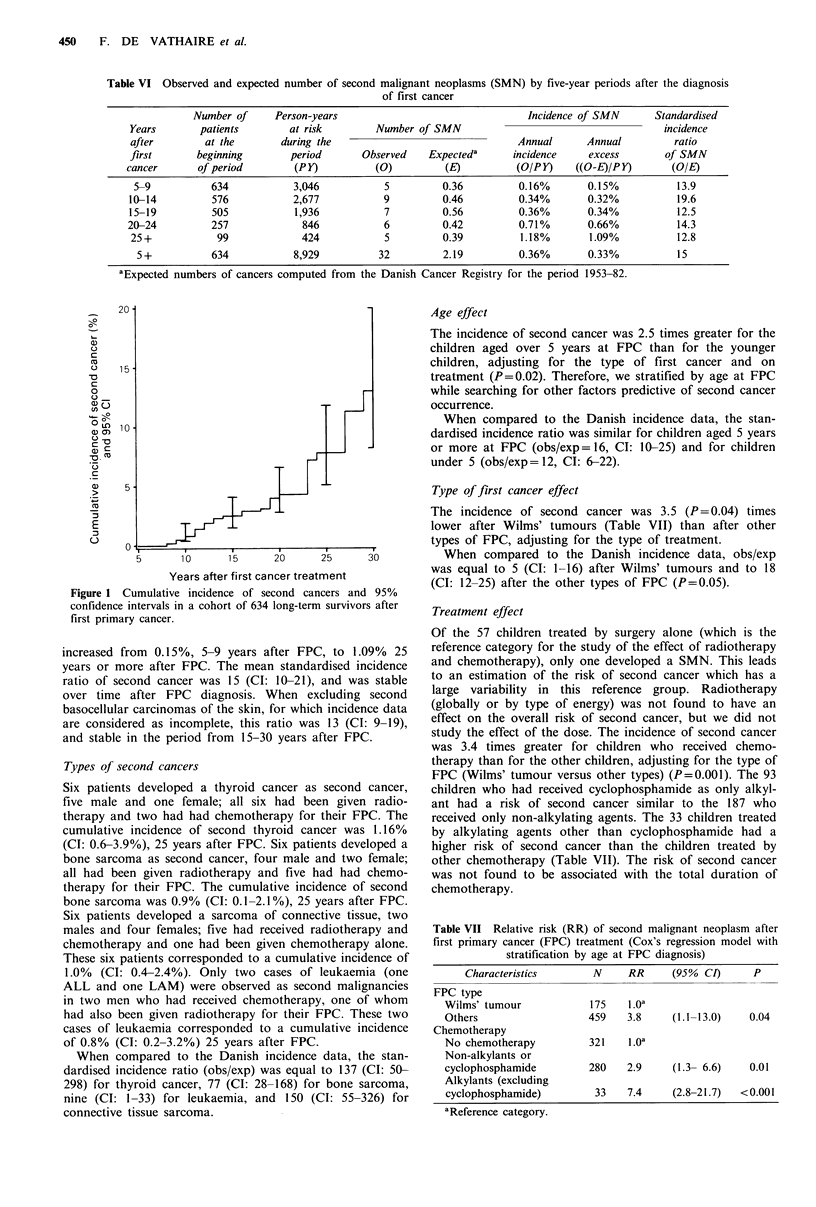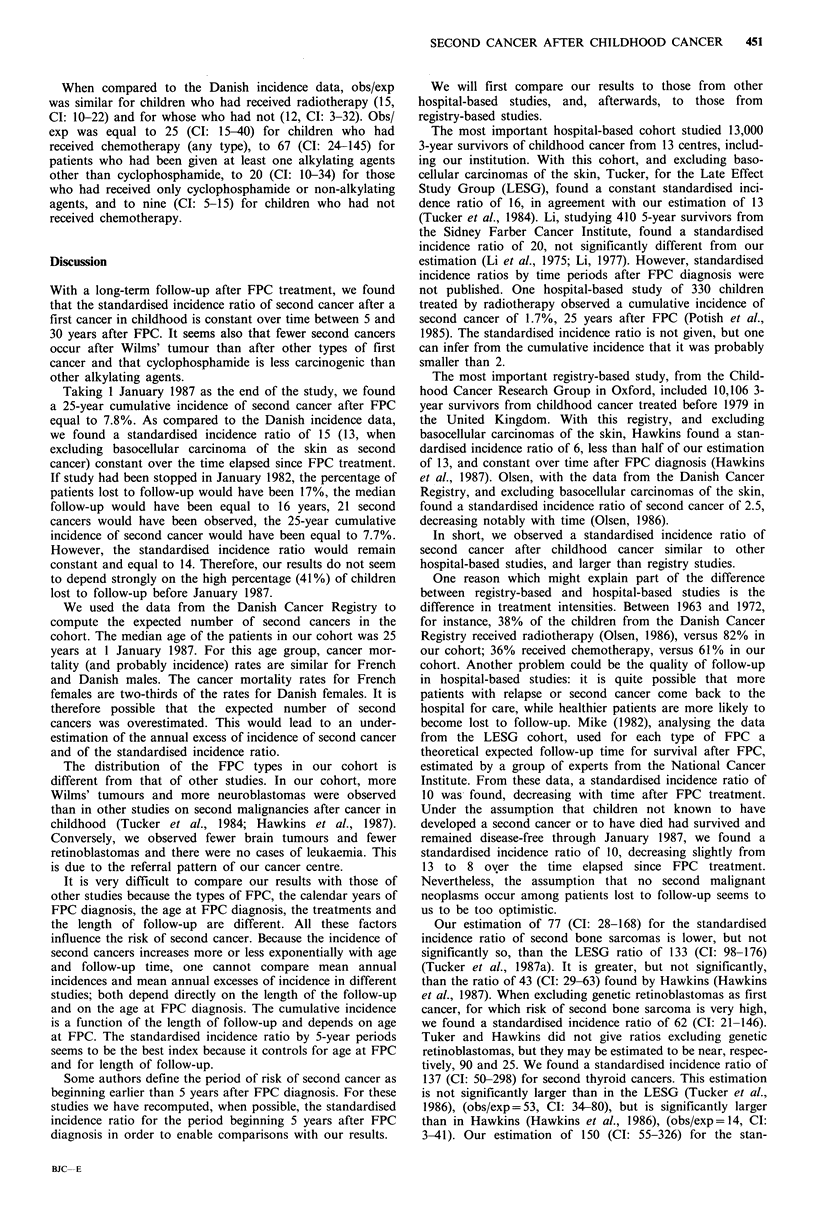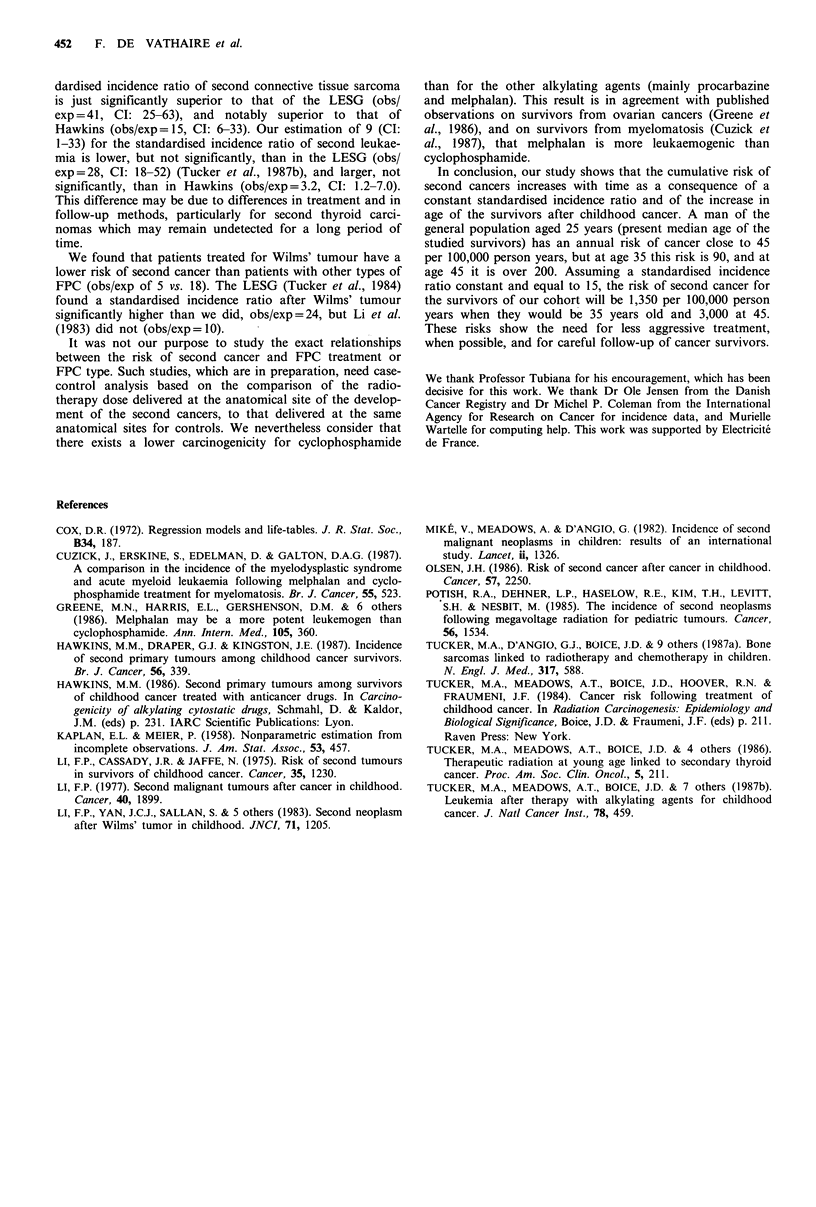# Long-term risk of second malignant neoplasm after a cancer in childhood.

**DOI:** 10.1038/bjc.1989.92

**Published:** 1989-03

**Authors:** F. de Vathaire, O. Schweisguth, C. Rodary, P. FranÃ§ois, D. Sarrazin, O. Oberlin, C. Hill, M. A. Raquin, A. Dutreix, R. Flamant

**Affiliations:** DÃ©partement de Statistique MÃ©dicale, Institut Gustave Roussy, Villejuif, France.

## Abstract

The risk of subsequent second malignant neoplasm was studied in a cohort of 634 patients, treated for a childhood cancer at the Gustave Roussy Institute between 1942 and 1969, and in complete remission five years after diagnosis. The most frequent types of first primary cancers (FPC) were Wilms' tumours (28% of the children), neuroblastomas (16%), lymphomas (12%) and soft tissue sarcomas (11%). Median follow-up duration after FPC was 19 years. Thirty-two patients (obs = 32) developed a total of 35 second cancers. Bone, thyroid, connective tissues and skin were the most frequent types of second cancer, with six patients for each type. The average annual incidence of second cancer was 0.36%. The average annual incidence for the periods 5-9, 10-14, 15-19, 20-24 and 25+ years after FPC was respectively 0.16%, 0.34%, 0.36%, 0.71% and 1.18%. The cumulative incidence of second cancer for the periods 5-20, 5-25 and 5-30 years after FPC was, respectively, 4.3% (95% CI: 2.8-6.6%), 7.8% (95% CI: 5.1-11.8%) and 13.0% (95% CI: 8.2-20.0%). The expected number of cancers in the cohort, computed from Danish cancer incidence data, was exp = 2.2. When compared to this expected number, the average annual excess incidence of second cancer, defined as obs-exp divided by the number of person years of observation, was 0.33%. This rose from 0.15% for the period 5-9 years after FPC to 1.09% for the period beginning 25 years after FPC. The standardised incidence ratio of second cancer (i.e. obs/exp) was 15 (95% CI: 10-21), and was fairly constant in the period extending from 15 to 20 years after FPC diagnosis. Obs/exp was equal to 25 for the patients who had had chemotherapy and equal to 9 for those who had not. Cyclophosphamide seemed less carcinogenic than the other alkylating agents. Obs/exp was similar for the patients who had received radiotherapy and for those who had not. The risk of cancer increased with age in the reference population and increased faster in the cohort, because the standardised incidence ratio is constant over a long period.


*Br. J. Cancer* (1989), **59**, 448–452 © The Macmillan Press Ltd., 1989

### Long-term risk of second malignant neoplasm after a cancer in childhood

F. de Vathaire[1], O. Schweisguth[2], C. Rodary[1], P. François[3], D. Sarrazin[4], O. Oberlin[2], C. Hill[1], M.A. Raquin[2], A. Dutreix[3] & R. Flamant[1]

[1]*Département de Statistique Médicale;* [2]*Département de Pédiatrie;* [3]*Département de Radiophysique and* [4]*Département de Radiothérapie, Institut Gustave Roussy, Rue Camille Desmoulins, 94805 Villejuif Cedex, France.*

**Summary** The risk of subsequent second malignant neoplasm was studied in a cohort of 634 patients, treated for a childhood cancer at the Gustave Roussy Institute between 1942 and 1969, and in complete remission five years after diagnosis. The most frequent types of first primary cancers (FPC) were Wilms' tumours (28% of the children), neuroblastomas (16%), lymphomas (12%) and soft tissue sarcomas (11%). Median follow-up duration after FPC was 19 years. Thirty-two patients (obs = 32) developed a total of 35 second cancers. Bone, thyroid, connective tissues and skin were the most frequent types of second cancer, with six patients for each type. The average annual incidence of second cancer was 0.36%. The average annual incidence for the periods 5–9, 10–14, 15–19, 20–24 and 25+ years after FPC was respectively 0.16%, 0.34%, 0.36%, 0.71% and 1.18%. The cumulative incidence of second cancer for the periods 5–20, 5–25 and 5–30 years after FPC was, respectively, 4.3% (95% CI: 2.8–6.6%), 7.8% (95% CI: 5.1–11.8%) and 13.0% (95% CI: 8.2–20.0%). The expected number of cancers in the cohort, computed from Danish cancer incidence data, was exp = 2.2. When compared to this expected number, the average annual excess incidence of second cancer, defined as obs − exp divided by the number of person years of observation, was 0.33%. This rose from 0.15% for the period 5–9 years after FPC to 1.09% for the period beginning 25 years after FPC. The standardised incidence ratio of second cancer (i.e. obs/exp) was 15 (95% CI: 10–21), and was fairly constant in the period extending from 15 to 20 years after FPC diagnosis. Obs/exp was equal to 25 for the patients who had had chemotherapy and equal to 9 for those who had not. Cyclophosphamide seemed less carcinogenic than the other alkylating agents. Obs/exp was similar for the patients who had received radiotherapy and for those who had not. The risk of cancer increased with age in the reference population and increased faster in the cohort, because the standardised incidence ratio is constant over a long period.

Survivors of childhood cancer began to appear in the early 1940s and the proportion of long-term survivors has regularly increased during the past 30 years. This improvement is due to more intensive therapies. However, these more intensive therapies may increase the risk of later complications such as infertility, growth disturbances, deformities and second primary malignant neoplasms.

The published results on the variation of the incidence of second cancers with the time elapsed after first primary cancer (FPC) treatment are contradictory. Estimation of the 25 years cumulative incidence of second cancer after childhood cancer varies from 1.7% (Potish *et al.*, 1985) to 12.1% (Tucker *et al.*, 1984).

The present work reports the results of a cohort study designed to evaluate the long-term risk of cancer according to the time elapsed after FPC. This study was possible because all the children seen between 1942 and 1969 at the Gustave Roussy Institute (IGR) were treated and followed actively by one of us (O.S.).

### Materials and methods

### *Patients*

The study included all of the 634 patients who were treated at the IGR between 1942 and 1969 for a cancer in childhood (age under 17), and who were alive and free of disease five years after cancer diagnosis. The children who died or those with active disease at five years after FPC diagnosis have not been included because the risk of death from the first cancer is very high during this period. None of these excluded patients had developed a second malignancy neoplasm. Information on treatment was abstracted from medical and technical records of IGR. The diagnoses of first and second malignant neoplasms were confirmed by histology, cytology or tumours markers, in the great majority, or by review of medical records when a tumour sample was not available (brain tumours, for instance). Histiocytosis and fibromatosis were not considered as cancer.

Characteristics of the cohort are reported in Table I. Among the 634 patients, 70 developed distant metastasis, 146 had loco-regional relapses and two had both metastasis and loco-regional relapses during the 5 years after diagnosis of the first cancer. By definition of the cohort, all were free of disease five years after FPC diagnosis. Table II shows that the most frequent types of first cancer were Wilms' tumour (28%) and neuroblastoma (16%). Table III shows that nearly 80% of the children received radiotherapy and half received chemotherapy. Details of the chemotherapy and radiotherapy are given in Table IV and in Table V, respectively.

### *Statistical methods*

The period of risk of a second cancer was defined as beginning five years after the diagnosis of the first cancer and ending at the time of the first of the following events: occurrence of second cancer, death, loss to follow-up or 1

**Table I** Characteristics of the 634 patients of the cohort

| *Characteristics* | |
|---|---|
| Calendar year of first cancer treatment: median (range) | 1965 (1942–69) |
| Sex ratio (M/F) | 1.07 |
| Age at first cancer treatment in years: median (range) | 3 (0–17) |
| Years of follow-up after first cancer diagnosis: median (range) | 19 (5–43) |
| Number of patients with second primary cancers | 32 |
| Delay between first and second cancer in years: median (range) | 17 (7–31) |

Correspondence: F. de Vathaire.
Received 25 June 1988, and in revised form, 2 November 1988.

**Table II** Number of second malignant neoplasms by type of first cancer

| *Type of first cancer (number of patients)* | | *Second malignant neoplasms* | | | | | | | | |
|---|---|---|---|---|---|---|---|---|---|---|
| | | *Bone* | *Thyroid* | *Connective tissue* | *Brain* | *Leukaemia* | *Breast* | *Skin* | *Others* | *Total* |
| Wilms' tumour | (175) | 1 | – | 1 | 1 | – | – | – | – | 3 |
| Neuroblastoma | (99) | – | 3 | 1 | – | – | 1 | – | 2 | 7 |
| Soft tissue sarcoma | (71) | 1 | 1 | 2 | 1 | – | – | 1 | – | 6 |
| Lymphoma | | | | | | | | | | |
| Hodgkin's Disease | (39) | – | – | 1 | – | 1 | 1 | – | 2 | 5 |
| Other lymphomas | (34) | 1 | 1 | – | – | – | – | – | – | 2 |
| Bone | | | | | | | | | | |
| Ewing's sarcoma | (21) | 2 | – | 1 | – | – | – | – | – | 3 |
| Osteogenic sarcoma | (18) | – | – | – | – | – | – | – | – | 0 |
| Brain tumour | (81) | – | 1 | – | 1 | – | – | 2 | – | 4 |
| Gonadal tumour | (29) | – | – | – | – | – | – | – | – | 0 |
| Thyroid carcinoma | (22) | – | – | – | – | – | – | 1 | – | 1 |
| Retinoblastoma | | | | | | | | | | |
| Unilateral retin. | (10) | – | – | – | – | – | – | – | – | 0 |
| Bilateral retin. | (10) | 1 | – | – | – | – | – | – | – | 1 |
| Others | (25) | – | – | – | – | 1 | 1 | – | 1 | 3 |
| Total | (634) | 6 | 6 | 6 | 3 | 2 | 3 | 4 | 5 | 35[a] |

[a]Three patients developed two second cancers: a thyroid carcinoma and an undifferentiated sarcoma of the mediastin after a rhabdomyosarcoma as FPC, a carcinoma of the parotid and a breast phyllod sarcoma after a Hodgkin's disease as FPC, and two basocellular carcinomas of the skin after a ganglio-neuroblastoma as FPC.

**Table III** Treatment received by the 634 children for the first primary cancer (number of children treated by surgery for each category)

| | | *Radiotherapy* | | |
|---|---|---|---|---|
| | | *No* | *Yes* | *Total* |
| Chemotherapy | No | 57 (57) | 264 (191) | 321 (248) |
| | Yes | 49 (45) | 264 (219) | 313 (264) |
| | Total | 106 (102) | 528 (410) | 634 (512) |

**Table IV** Description of chemotherapies administrated for first primary cancer

| *Type of drug* | *Number of children*[a] | *Dose* $(mg\,m^{-2})$ *Median* | *(Range)* |
|---|---|---|---|
| Any alkylating agent | 126 | | |
| Cyclophosphamide | 101 | 4,200 | (250–70,000) |
| Procarbazine | 22 | 6,350 | (800–27,000) |
| Melphalan | 8 | 100 | (45–1,800) |
| Other alkylants | 6 | – | |
| Any non-alkylating agents | 225 | | |
| Dactinomycin | 164 | 2.7 | (0.1–26.7) |
| Vincristin | 112 | 12 | (1–57) |
| Methotrexate | 27 | 560 | (933–4,500) |
| Vinblastin | 23 | 138 | (24–662) |
| Nitrogen mustard | 18 | 72 | (8–154) |
| Other non-alkylants | 30 | | – |
| Any drugs | 313 | | – |

[a]132 children have received more than one drug; [b]$IU\,m^{-2}$.

**Table V** Type of radiotherapy for first primary cancer

| *Type of energy* | *Number of children*[a] |
|---|---|
| Orthovoltage | 250 |
| Cobalt-60 | 172 |
| High energy X-rays | 101 |
| Electrons | 37 |
| Brachytherapy | 16 |
| Unknown | 9 |
| Any type of radiotherapy | 528 |

[a]Fifty-five children were treated by more than one type of energy.

January 1987. The cumulative incidence of second cancer was estimated by the Kaplan–Meier method (Kaplan & Meier, 1958). Prognostic factors of second cancer occurrence were identified using the Cox regression model (Cox, 1972). Since there is no national cancer registry in France and since regional registries contain no incidence data before 1975, expected numbers of cancer by sex, age and calendar year were obtained from the Danish Cancer Registry for the period 1953–82. The excess of incidence was computed as the difference between the observed number of second cancer (obs) and the expected number (exp), divided by the number of person-years of observation (PY). The standardised incidence ratio of second cancer was defined as the ratio between the observed and the expected numbers of cancers (obs/exp). Confidence intervals of the standardised incidence ratios were calculated using exact Poisson probabilities. All confidence intervals (CI) reported in this work are 95% confidence intervals.

### Results

A total of 107 patients (17%) were lost to follow-up before January 1982 and 263 patients (41%) were lost before January 1987. Median follow-up after the diagnosis of the first cancer was 19 years (range 5–43).

Thirty-five second cancers were observed in 32 patients. The most frequent sites of second cancers were bone, thyroid, connective tissue and skin (Table II). Among the six second cancers of the skin, one was a malignant melanoma, the five others being basocellular carcinomas.

### *Overall incidence of second cancers*

The mean annual incidence of second cancer was 7 times greater 25 years or more after FPC diagnosis than 5–9 years after FPC (i.e. 1.18%/0.16%) (Table VI). The cumulative incidence of second cancer was 4.3% (CI: 2.8–6.6%) 20 years after FPC diagnosis, 7.8% (CI: 5.1–11.8%) 25 years after, and 13.0% (CI: 8.2–20.0%) 30 years after (Figure 1). Excluding the five second basocellular carcinomas of the skin, these rates are, respectively, 4.2, 6.7 and 9.6%. Neither FPC related events (metastases or locoregional relapses) nor sex were found to have a significant influence on the risk of second cancer.

From the Danish cancer incidence data, 2.2 cancers were expected in the cohort during the follow-up period (Table VI). The annual excess of incidence of second cancer

**Table VI** Observed and expected number of second malignant neoplasms (SMN) by five-year periods after the diagnosis of first cancer

| *Years after first cancer* | *Number of patients at the beginning of period* | *Person-years at risk during the period (PY)* | *Number of SMN* | | *Incidence of SMN* | | *Standardised incidence ratio of SMN (O/E)* |
|---|---|---|---|---|---|---|---|
| | | | *Observed (O)* | *Expected*[a] *(E)* | *Annual incidence (O/PY)* | *Annual excess ((O-E)/PY)* | |
| 5–9 | 634 | 3,046 | 5 | 0.36 | 0.16% | 0.15% | 13.9 |
| 10–14 | 576 | 2,677 | 9 | 0.46 | 0.34% | 0.32% | 19.6 |
| 15–19 | 505 | 1,936 | 7 | 0.56 | 0.36% | 0.34% | 12.5 |
| 20–24 | 257 | 846 | 6 | 0.42 | 0.71% | 0.66% | 14.3 |
| 25+ | 99 | 424 | 5 | 0.39 | 1.18% | 1.09% | 12.8 |
| 5+ | 634 | 8,929 | 32 | 2.19 | 0.36% | 0.33% | 15 |

[a]Expected numbers of cancers computed from the Danish Cancer Registry for the period 1953–82.

**Figure 1** Cumulative incidence of second cancers and 95% confidence intervals in a cohort of 634 long-term survivors after first primary cancer.

increased from 0.15%, 5–9 years after FPC, to 1.09% 25 years or more after FPC. The mean standardised incidence ratio of second cancer was 15 (CI: 10–21), and was stable over time after FPC diagnosis. When excluding second basocellular carcinomas of the skin, for which incidence data are considered as incomplete, this ratio was 13 (CI: 9–19), and stable in the period from 15–30 years after FPC.

*Types of second cancers*

Six patients developed a thyroid cancer as second cancer, five male and one female; all six had been given radiotherapy and two had had chemotherapy for their FPC. The cumulative incidence of second thyroid cancer was 1.16% (CI: 0.6–3.9%), 25 years after FPC. Six patients developed a bone sarcoma as second cancer, four male and two female; all had been given radiotherapy and five had had chemotherapy for their FPC. The cumulative incidence of second bone sarcoma was 0.9% (CI: 0.1–2.1%), 25 years after FPC. Six patients developed a sarcoma of connective tissue, two males and four females; five had received radiotherapy and chemotherapy and one had been given chemotherapy alone. These six patients corresponded to a cumulative incidence of 1.0% (CI: 0.4–2.4%). Only two cases of leukaemia (one ALL and one LAM) were observed as second malignancies in two men who had received chemotherapy, one of whom had also been given radiotherapy for their FPC. These two cases of leukaemia corresponded to a cumulative incidence of 0.8% (CI: 0.2–3.2%) 25 years after FPC.

When compared to the Danish incidence data, the standardised incidence ratio (obs/exp) was equal to 137 (CI: 50–298) for thyroid cancer, 77 (CI: 28–168) for bone sarcoma, nine (CI: 1–33) for leukaemia, and 150 (CI: 55–326) for connective tissue sarcoma.

*Age effect*

The incidence of second cancer was 2.5 times greater for the children aged over 5 years at FPC than for the younger children, adjusting for the type of first cancer and on treatment ($P=0.02$). Therefore, we stratified by age at FPC while searching for other factors predictive of second cancer occurrence.

When compared to the Danish incidence data, the standardised incidence ratio was similar for children aged 5 years or more at FPC (obs/exp = 16, CI: 10–25) and for children under 5 (obs/exp = 12, CI: 6–22).

*Type of first cancer effect*

The incidence of second cancer was 3.5 ($P=0.04$) times lower after Wilms' tumours (Table VII) than after other types of FPC, adjusting for the type of treatment.

When compared to the Danish incidence data, obs/exp was equal to 5 (CI: 1–16) after Wilms' tumours and to 18 (CI: 12–25) after the other types of FPC ($P=0.05$).

*Treatment effect*

Of the 57 children treated by surgery alone (which is the reference category for the study of the effect of radiotherapy and chemotherapy), only one developed a SMN. This leads to an estimation of the risk of second cancer which has a large variability in this reference group. Radiotherapy (globally or by type of energy) was not found to have an effect on the overall risk of second cancer, but we did not study the effect of the dose. The incidence of second cancer was 3.4 times greater for children who received chemotherapy than for the other children, adjusting for the type of FPC (Wilms' tumour versus other types) ($P=0.001$). The 93 children who had received cyclophosphamide as only alkylant had a risk of second cancer similar to the 187 who received only non-alkylating agents. The 33 children treated by alkylating agents other than cyclophosphamide had a higher risk of second cancer than the children treated by other chemotherapy (Table VII). The risk of second cancer was not found to be associated with the total duration of chemotherapy.

**Table VII** Relative risk (RR) of second malignant neoplasm after first primary cancer (FPC) treatment (Cox's regression model with stratification by age at FPC diagnosis)

| *Characteristics* | *N* | *RR* | *(95% CI)* | *P* |
|---|---|---|---|---|
| FPC type | | | | |
| Wilms' tumour | 175 | 1.0[a] | | |
| Others | 459 | 3.8 | (1.1–13.0) | 0.04 |
| Chemotherapy | | | | |
| No chemotherapy | 321 | 1.0[a] | | |
| Non-alkylants or cyclophosphamide | 280 | 2.9 | (1.3– 6.6) | 0.01 |
| Alkylants (excluding cyclophosphamide) | 33 | 7.4 | (2.8–21.7) | <0.001 |

[a]Reference category.

When compared to the Danish incidence data, obs/exp was similar for children who had received radiotherapy (15, CI: 10–22) and for whose who had not (12, CI: 3–32). Obs/exp was equal to 25 (CI: 15–40) for children who had received chemotherapy (any type), to 67 (CI: 24–145) for patients who had been given at least one alkylating agents other than cyclophosphamide, to 20 (CI: 10–34) for those who had received only cyclophosphamide or non-alkylating agents, and to nine (CI: 5–15) for children who had not received chemotherapy.

### Discussion

With a long-term follow-up after FPC treatment, we found that the standardised incidence ratio of second cancer after a first cancer in childhood is constant over time between 5 and 30 years after FPC. It seems also that fewer second cancers occur after Wilms' tumour than after other types of first cancer and that cyclophosphamide is less carcinogenic than other alkylating agents.

Taking 1 January 1987 as the end of the study, we found a 25-year cumulative incidence of second cancer after FPC equal to 7.8%. As compared to the Danish incidence data, we found a standardised incidence ratio of 15 (13, when excluding basocellular carcinoma of the skin as second cancer) constant over the time elapsed since FPC treatment. If study had been stopped in January 1982, the percentage of patients lost to follow-up would have been 17%, the median follow-up would have been equal to 16 years, 21 second cancers would have been observed, the 25-year cumulative incidence of second cancer would have been equal to 7.7%. However, the standardised incidence ratio would remain constant and equal to 14. Therefore, our results do not seem to depend strongly on the high percentage (41%) of children lost to follow-up before January 1987.

We used the data from the Danish Cancer Registry to compute the expected number of second cancers in the cohort. The median age of the patients in our cohort was 25 years at 1 January 1987. For this age group, cancer mortality (and probably incidence) rates are similar for French and Danish males. The cancer mortality rates for French females are two-thirds of the rates for Danish females. It is therefore possible that the expected number of second cancers was overestimated. This would lead to an underestimation of the annual excess of incidence of second cancer and of the standardised incidence ratio.

The distribution of the FPC types in our cohort is different from that of other studies. In our cohort, more Wilms' tumours and more neuroblastomas were observed than in other studies on second malignancies after cancer in childhood (Tucker *et al.*, 1984; Hawkins *et al.*, 1987). Conversely, we observed fewer brain tumours and fewer retinoblastomas and there were no cases of leukaemia. This is due to the referral pattern of our cancer centre.

It is very difficult to compare our results with those of other studies because the types of FPC, the calendar years of FPC diagnosis, the age at FPC diagnosis, the treatments and the length of follow-up are different. All these factors influence the risk of second cancer. Because the incidence of second cancers increases more or less exponentially with age and follow-up time, one cannot compare mean annual incidences and mean annual excesses of incidence in different studies; both depend directly on the length of the follow-up and on the age at FPC diagnosis. The cumulative incidence is a function of the length of follow-up and depends on age at FPC. The standardised incidence ratio by 5-year periods seems to be the best index because it controls for age at FPC and for length of follow-up.

Some authors define the period of risk of second cancer as beginning earlier than 5 years after FPC diagnosis. For these studies we have recomputed, when possible, the standardised incidence ratio for the period beginning 5 years after FPC diagnosis in order to enable comparisons with our results.

We will first compare our results to those from other hospital-based studies, and, afterwards, to those from registry-based studies.

The most important hospital-based cohort studied 13,000 3-year survivors of childhood cancer from 13 centres, including our institution. With this cohort, and excluding basocellular carcinomas of the skin, Tucker, for the Late Effect Study Group (LESG), found a constant standardised incidence ratio of 16, in agreement with our estimation of 13 (Tucker *et al.*, 1984). Li, studying 410 5-year survivors from the Sidney Farber Cancer Institute, found a standardised incidence ratio of 20, not significantly different from our estimation (Li *et al.*, 1975; Li, 1977). However, standardised incidence ratios by time periods after FPC diagnosis were not published. One hospital-based study of 330 children treated by radiotherapy observed a cumulative incidence of second cancer of 1.7%, 25 years after FPC (Potish *et al.*, 1985). The standardised incidence ratio is not given, but one can infer from the cumulative incidence that it was probably smaller than 2.

The most important registry-based study, from the Childhood Cancer Research Group in Oxford, included 10,106 3-year survivors from childhood cancer treated before 1979 in the United Kingdom. With this registry, and excluding basocellular carcinomas of the skin, Hawkins found a standardised incidence ratio of 6, less than half of our estimation of 13, and constant over time after FPC diagnosis (Hawkins *et al.*, 1987). Olsen, with the data from the Danish Cancer Registry, and excluding basocellular carcinomas of the skin, found a standardised incidence ratio of second cancer of 2.5, decreasing notably with time (Olsen, 1986).

In short, we observed a standardised incidence ratio of second cancer after childhood cancer similar to other hospital-based studies, and larger than registry studies.

One reason which might explain part of the difference between registry-based and hospital-based studies is the difference in treatment intensities. Between 1963 and 1972, for instance, 38% of the children from the Danish Cancer Registry received radiotherapy (Olsen, 1986), versus 82% in our cohort; 36% received chemotherapy, versus 61% in our cohort. Another problem could be the quality of follow-up in hospital-based studies: it is quite possible that more patients with relapse or second cancer come back to the hospital for care, while healthier patients are more likely to become lost to follow-up. Mike (1982), analysing the data from the LESG cohort, used for each type of FPC a theoretical expected follow-up time for survival after FPC, estimated by a group of experts from the National Cancer Institute. From these data, a standardised incidence ratio of 10 was found, decreasing with time after FPC treatment. Under the assumption that children not known to have developed a second cancer or to have died had survived and remained disease-free through January 1987, we found a standardised incidence ratio of 10, decreasing slightly from 13 to 8 over the time elapsed since FPC treatment. Nevertheless, the assumption that no second malignant neoplasms occur among patients lost to follow-up seems to us to be too optimistic.

Our estimation of 77 (CI: 28–168) for the standardised incidence ratio of second bone sarcomas is lower, but not significantly so, than the LESG ratio of 133 (CI: 98–176) (Tucker *et al.*, 1987a). It is greater, but not significantly, than the ratio of 43 (CI: 29–63) found by Hawkins (Hawkins *et al.*, 1987). When excluding genetic retinoblastomas as first cancer, for which risk of second bone sarcoma is very high, we found a standardised incidence ratio of 62 (CI: 21–146). Tuker and Hawkins did not give ratios excluding genetic retinoblastomas, but they may be estimated to be near, respectively, 90 and 25. We found a standardised incidence ratio of 137 (CI: 50–298) for second thyroid cancers. This estimation is not significantly larger than in the LESG (Tucker *et al.*, 1986), (obs/exp = 53, CI: 34–80), but is significantly larger than in Hawkins (Hawkins *et al.*, 1986), (obs/exp = 14, CI: 3–41). Our estimation of 150 (CI: 55–326) for the standardised incidence ratio of second connective tissue sarcoma is just significantly superior to that of the LESG (obs/exp = 41, CI: 25–63), and notably superior to that of Hawkins (obs/exp = 15, CI: 6–33). Our estimation of 9 (CI: 1–33) for the standardised incidence ratio of second leukaemia is lower, but not significantly, than in the LESG (obs/exp = 28, CI: 18–52) (Tucker *et al.*, 1987b), and larger, not significantly, than in Hawkins (obs/exp = 3.2, CI: 1.2–7.0). This difference may be due to differences in treatment and in follow-up methods, particularly for second thyroid carcinomas which may remain undetected for a long period of time.

We found that patients treated for Wilms' tumour have a lower risk of second cancer than patients with other types of FPC (obs/exp of 5 *vs.* 18). The LESG (Tucker *et al.*, 1984) found a standardised incidence ratio after Wilms' tumour significantly higher than we did, obs/exp = 24, but Li *et al.* (1983) did not (obs/exp = 10).

It was not our purpose to study the exact relationships between the risk of second cancer and FPC treatment or FPC type. Such studies, which are in preparation, need case-control analysis based on the comparison of the radiotherapy dose delivered at the anatomical site of the development of the second cancers, to that delivered at the same anatomical sites for controls. We nevertheless consider that there exists a lower carcinogenicity for cyclophosphamide than for the other alkylating agents (mainly procarbazine and melphalan). This result is in agreement with published observations on survivors from ovarian cancers (Greene *et al.*, 1986), and on survivors from myelomatosis (Cuzick *et al.*, 1987), that melphalan is more leukaemogenic than cyclophosphamide.

In conclusion, our study shows that the cumulative risk of second cancers increases with time as a consequence of a constant standardised incidence ratio and of the increase in age of the survivors after childhood cancer. A man of the general population aged 25 years (present median age of the studied survivors) has an annual risk of cancer close to 45 per 100,000 person years, but at age 35 this risk is 90, and at age 45 it is over 200. Assuming a standardised incidence ratio constant and equal to 15, the risk of second cancer for the survivors of our cohort will be 1,350 per 100,000 person years when they would be 35 years old and 3,000 at 45. These risks show the need for less aggressive treatment, when possible, and for careful follow-up of cancer survivors.

We thank Professor Tubiana for his encouragement, which has been decisive for this work. We thank Dr Ole Jensen from the Danish Cancer Registry and Dr Michel P. Coleman from the International Agency for Research on Cancer for incidence data, and Murielle Wartelle for computing help. This work was supported by Electricité de France.

### References

COX, D.R. (1972). Regression models and life-tables. *J. R. Stat. Soc.*, **B34**, 187.

CUZICK, J., ERSKINE, S., EDELMAN, D. & GALTON, D.A.G. (1987). A comparison in the incidence of the myelodysplastic syndrome and acute myeloid leukaemia following melphalan and cyclophosphamide treatment for myelomatosis. *Br. J. Cancer*, **55**, 523.

GREENE, M.N., HARRIS, E.L., GERSHENSON, D.M. & 6 others (1986). Melphalan may be a more potent leukemogen than cyclophosphamide. *Ann. Intern. Med.*, **105**, 360.

HAWKINS, M.M., DRAPER, G.J. & KINGSTON, J.E. (1987). Incidence of second primary tumours among childhood cancer survivors. *Br. J. Cancer*, **56**, 339.

HAWKINS, M.M. (1986). Second primary tumours among survivors of childhood cancer treated with anticancer drugs. In *Carcinogenicity of alkylating cytostatic drugs*, Schmahl, D. & Kaldor, J.M. (eds) p. 231. IARC Scientific Publications: Lyon.

KAPLAN, E.L. & MEIER, P. (1958). Nonparametric estimation from incomplete observations. *J. Am. Stat. Assoc.*, **53**, 457.

LI, F.P., CASSADY, J.R. & JAFFE, N. (1975). Risk of second tumours in survivors of childhood cancer. *Cancer*, **35**, 1230.

LI, F.P. (1977). Second malignant tumours after cancer in childhood. *Cancer*, **40**, 1899.

LI, F.P., YAN, J.C.J., SALLAN, S. & 5 others (1983). Second neoplasm after Wilms' tumor in childhood. *JNCI*, **71**, 1205.

MIKÉ, V., MEADOWS, A. & D'ANGIO, G. (1982). Incidence of second malignant neoplasms in children: results of an international study. *Lancet*, **ii**, 1326.

OLSEN, J.H. (1986). Risk of second cancer after cancer in childhood. *Cancer*, **57**, 2250.

POTISH, R.A., DEHNER, L.P., HASELOW, R.E., KIM, T.H., LEVITT, S.H. & NESBIT, M. (1985). The incidence of second neoplasms following megavoltage radiation for pediatric tumours. *Cancer*, **56**, 1534.

TUCKER, M.A., D'ANGIO, G.J., BOICE, J.D. & 9 others (1987a). Bone sarcomas linked to radiotherapy and chemotherapy in children. *N. Engl. J. Med.*, **317**, 588.

TUCKER, M.A., MEADOWS, A.T., BOICE, J.D., HOOVER, R.N. & FRAUMENI, J.F. (1984). Cancer risk following treatment of childhood cancer. In *Radiation Carcinogenesis: Epidemiology and Biological Significance*, Boice, J.D. & Fraumeni, J.F. (eds) p. 211. Raven Press: New York.

TUCKER, M.A., MEADOWS, A.T., BOICE, J.D. & 4 others (1986). Therapeutic radiation at young age linked to secondary thyroid cancer. *Proc. Am. Soc. Clin. Oncol.*, **5**, 211.

TUCKER, M.A., MEADOWS, A.T., BOICE, J.D. & 7 others (1987b). Leukemia after therapy with alkylating agents for childhood cancer. *J. Natl Cancer Inst.*, **78**, 459.